# Did Menzel Paint His Own Babinski Sign?

**DOI:** 10.3389/fneur.2013.00096

**Published:** 2013-07-24

**Authors:** Anamarija Kavčič

**Affiliations:** ^1^Diakonissenanstalt zu Flensburg, NeurologyFlensburg, Germany

Adolph von Menzel (1815–1905), one of the greatest German realistic painters of the nineteenth Century (1), painted his right foot from normal perspective with the great toe extended (Figure 1B). The other toes seem to be relaxed. There are no suggestions of foot deformity like pes cavus.

**Figure 1:**
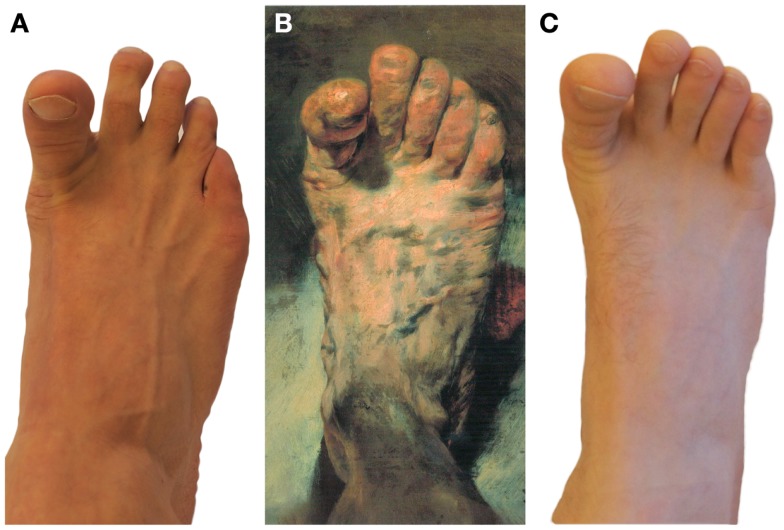
**(A)** A foot of a proband – a Babinski sign or a voluntary extension of a great toe? (A solution is found in the Supplementary material). **(B)** “The painter’s Foot” by Adolph von Menzel (1815–1905) (National Gallery Berlin). **(C)** A foot of another proband – a Babinski sign or a voluntary extension of a great toe? (A solution is found in the Supplementary material.)

Menzel finished that painting, oil on wood panel, in 1876 (1). He gave no official explanation, why he had chosen this motif. A son of his friend remembered that Menzel once, while taking a foot bath, suddenly looked at his feet and started to draw his own foot (2). He did it so intensively, that for a while he completely forgot about washing his feet (2).

Fried Michael, an American art critic and art historian, viewed the picture “The painter’s Foot” principally in its immense vitality (1). He assumed the odd extension of a great toe to be an imaginary manifestation of the body’s liveliness (1). Consequently, he believed, that the painter kept his great toe voluntarily extended as long and often as needed to finish that painting (1).

For a neurologist it is impossible to view that picture without thinking about the Babinski sign. The question – did Menzel paint, what he saw *per se* or did he paint, what he modeled just to make the picture dynamic – is asked more than a 100 years too late to be unambiguously answered by Menzel himself.

Menzel’s life and especially his work showed, that he was an outstanding observer and determined realist (1, 3). Being such a person, would he paint, what he did not see? In all probability not. He drew his hand as well and by doing this, he did not experiment with various finger positions (1). He just painted his hand in one of several normal postures.

If we therefore assume, that great toe extension, seen in his picture “The painter’s Foot” (Figure 1B) actually exhibits a reflex, i.e., the Babinski sign (4), we have to confront ourselves with another question. Did Menzel, a highly talented man, mentally and physically active almost to his death at the age of 89 years, i.e., in 1905 (1–3), have a neurological disorder at least from his early sixties on?

Considering his successful life, which was extraordinarily long for his time (1, 3), it seems implausible, that he would have had a significant neurological disorder. However, his medical case history was not completely irrelevant. Growing to a height of about 1.40 m (3), he was rather small in stature, but his parents and siblings were of normal height (3). As a child and also later he suffered from some kind of seizures (2, 3). According to scarce descriptions of these (2), it is to be supposed, that they were epileptic. They happened suddenly and assumingly without prodromes (2). Anyway, this disorder did not considerably restrict him when painting, even though for years he did not have an especially healthy lifestyle. He tended to work almost the whole day without resting or eating (2). Then late in the evening he would go to a pub and enjoyed a great meal (2). There is no indication, that he took any medicine regularly or that he ever injured himself because of a seizure (1–3). He did not smoke, but he did enjoy drinking wine (without being an alcoholic) (2). Interestingly, by painting he was able to use both his hands equally (3). As a matter of fact he started to paint with his left-hand, but over the course of time he learned to paint with his right hand (3).

Taking into account Menzel’s immense work – the best reflection of himself – and all the written memories of him, we can conclude, that Menzel quite likely painted, what he simply saw, i.e., his own Babinski sign and not a foot with one of many possible voluntary movements of a great toe (Figure 1B). Maybe water stimulated the sole of his foot while he was taking a foot bath and thus elicited the Babinski sign. However, Menzel had to notice instinctively the distinctiveness of the great toe extension. Why should he, one of the most prominent German artists of the nineteenth century, and very successful as well (1, 3), otherwise portray it?

## Supplementary Material

The Supplementary Material for this article can be found online at http://www.frontiersin.org/Neurology_Education/10.3389/fneur.2013.00096/full

Supplementary Figure S1 **(A solution of the questions, put in the Figure 1.) (A)** A voluntary extension of a great toe of a healthy physiotherapist. **(B)** “The painter’s Foot” by Adolph von Menzel (1815–1905) (National Gallery Berlin). **(C)** A Babinski sign in a patient with acute stroke.
