# Supplementary figures and images for: Did Menzel Paint His Own Babinski Sign?
Source: Front Neurol. 2013 Jul 24;4:96. doi: 10.3389/fneur.2013.00096 (PMC3721090; doi:10.3389/fneur.2013.00096)

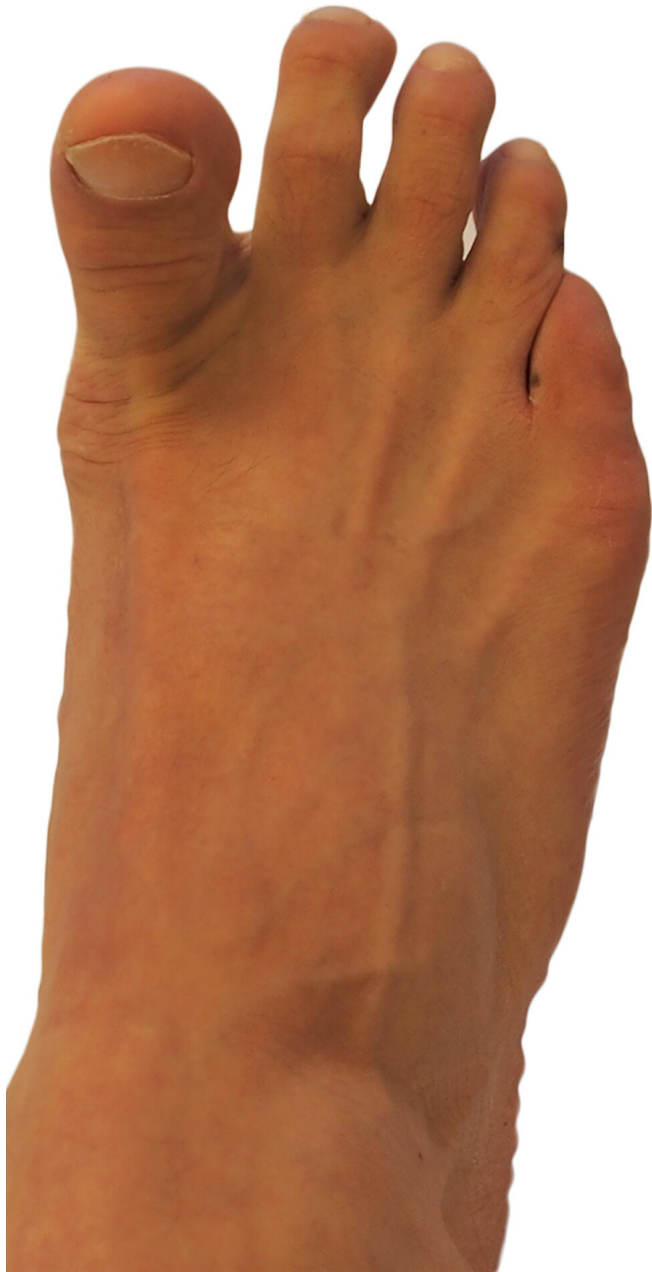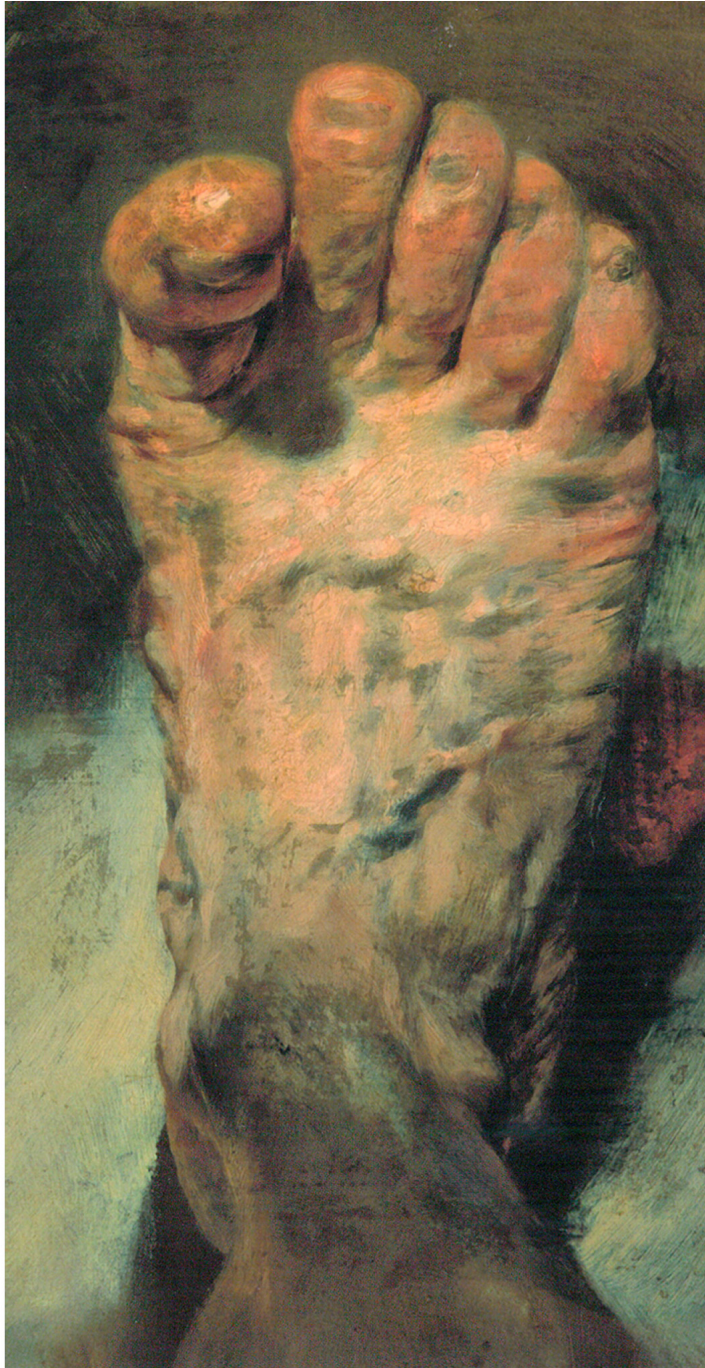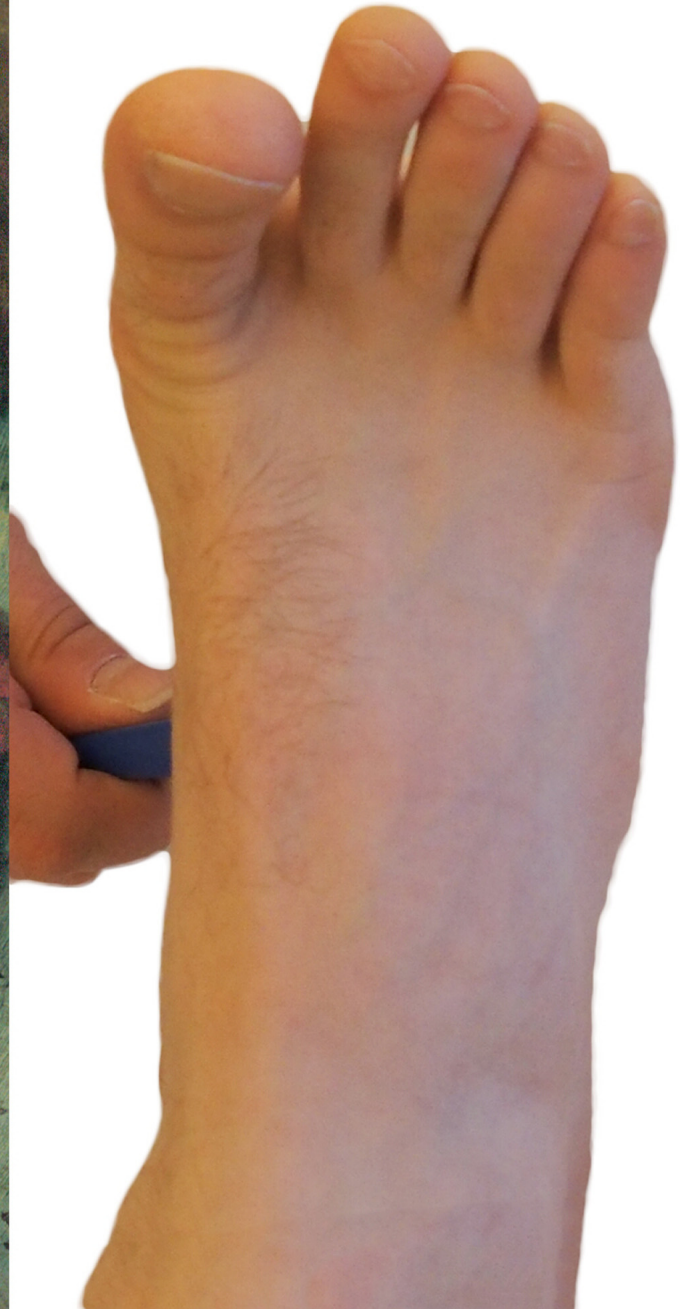

Supplement: Supplementary Figure S1 — (A solution of the questions, put in the Figure 1.) (A) A voluntary extension of a great toe of a healthy physiotherapist. (B) “The painter’s Foot” by Adolph von Menzel (1815–1905) (National Gallery Berlin). (C) A Babinski sign in a patient with acute stroke. [file 56745_Kavcic_Presentation1.PDF]
